# Higher consumption of ultra-processed foods is associated with disordered eating symptoms and low-quality diet in adults with obesity

**DOI:** 10.20945/2359-4292-2026-0010

**Published:** 2026-02-16

**Authors:** Carolina Machado Favaron, Marcos Mônico-Neto, Hanna Karen Moreira Antunes, Lia Rita Azeredo Bittencourt, Thales Delmondes Galvão, Sergio Tufik, Raquel Munhoz da Silveira Campos

**Affiliations:** 1 Programa de Pós-graduação Interdisciplinar em Ciências da Saúde, Universidade Federal de São Paulo, Santos, SP, Brasil; 2 BariMais — Medicina Integrada, São Paulo, SP, Brasil; 3 Programa de Pós-graduação em Psicobiologia, Universidade Federal de São Paulo, São Paulo, SP, Brasil

**Keywords:** Obesity, ultra-processed foods, feeding and eating disorders, binge eating disorder, bulimia

## Abstract

**Objective:**

This study aims to evaluate ultra-processed food consumption and eating behavior in adults with obesity.

**Subjects and methods:**

A cross-sectional study with 77 volunteers from São Paulo, Brazil. Food consumption was assessed using three 24-hour dietary recalls, classified by using the NOVA classification system, and the Diet Quality Index was also evaluated. Eating behavior and symptoms of binge eating and bulimia were assessed using the Bulimic Investigatory Test Edinburgh (BITE), the Dutch Eating Behaviour Questionnaire (DEBQ), and the Three Factor Eating Questionnaire (TFEQ-21).

**Results:**

The average BMI of the sample was 39.14 kg/m² ± 5.57, and the median caloric intake was 1661 kcal (756.07–4774.40), with a macronutrient distribution of 48% carbohydrates, 32% fat, and 20% protein. Volunteers were divided into tertiles of calories ingested from ultra-processed foods (%): 1^st^ < 24.10% (n = 25); 2^nd^ between 24.10%–35.40% (n = 26); and the 3^rd^ > 35.40% (n = 26). The sample showed intermediate diet quality (43.08 ± 10.17), while the 3rd tertile presented a low-quality diet (37 ± 10), differing from other groups (p = 0.001; p = 0.003). All groups showed intermediate BITE scores (19,6 ± 9,8), an indicator of unusual eating behavior. The third tertile had a higher symptom score than the first tertile (p = 0.008). In the association analysis, the consumption of ultra-processed foods was positively associated with the presence of binge eating and bulimia symptoms (p = 0.018), emotional (p = 0.001) and external eating (p = 0.001) as assessed by the DEBQ, and emotional (p = 0.008) and uncontrolled eating (p = 0.006) as assessed by the TFEQ-21. In contrast, diet quality was negatively associated with the consumption of ultra-processed foods (p < 0.001).

**Conclusion:**

Our findings suggest that higher consumption of ultra-processed foods by volunteers with obesity may be associated with higher scores for unusual eating behavior, symptoms of binge eating, and bulimia, in addition to augmented emotional, external and uncontrolled eating, and lower diet quality scores.

## INTRODUCTION

Nutritional transition is known to occur primarily in developing countries due to social, cultural, demographic, and economic factors. It is characterized by a reduction in infectious diseases and malnutrition cases, and an increase in obesity and non-communicable diseases, particularly driven by changes in dietary patterns with an increased consumption of foods rich in fats, especially of animal origin, sugar, and processed foods ^(1-3)^.

Currently, these foods can be defined by their degree of processing using the NOVA classification system, which categorizes ultra-processed foods as formulations prepared via various industrial techniques and the inclusion of food additives to increasing palatability, such as high-fructose corn syrup and hydrogenated oils, contributing to high fat and sugar content in these products ^(4)^.

High consumption of these foods has been associated with an increased prevalence of overweight and obesity ^(5-8)^, cardiovascular and cerebrovascular diseases ^(7,8)^, type 2 diabetes mellitus ^(9)^, metabolic syndrome ^(7,8,10)^, depression ^(7,8,11)^, anxiety ^(11)^, and a higher risk of all-cause mortality ^(7,8,12,13)^. Additionally, there is also evidence of these foods having the capacity to induce changes in eating behavior, triggering compulsive overeating ^(14)^.

Eating disorders can occur in individuals with obesity and is associated with a higher risk of social and health implications, in addition to compromising the outcome of interventions focused on body weight management ^(15,16)^. The etiology of eating disorders is multifactorial, involving environmental influences, such as body dissatisfaction and family stress, along with biological factors, such as hormonal changes in the neuroendocrine control of food consumption ^(17)^.

Recently, the association between the consumption of ultra-processed foods and the risk of symptoms related to eating disorders has been investigated ^(18)^. It is believed that the consumption of ultra-processed foods may trigger changes in endocrine and neurobiological pathways that regulate eating behavior, compromising conditions related to eating disorders. It is suggested that guidance on the exclusion of these foods could contribute to the effectiveness of conventional obesity treatment ^(19,20)^. To test this hypothesis, a cross-sectional study was conducted to assess the consumption of ultra-processed foods and the presence of eating disorder symptoms in individuals with obesity, as well as to improve the understanding of the factors that may worsen obesity and its comorbidities, and to support the development of more effective interventions that consider food processing and diet quality in obesity treatment.

## SUBJECTS AND METHODS

### Population

The volunteers were recruited via social media and from a private health service specializing in the clinical and surgical treatment of obesity, located in the city of São Paulo, SP, Brazil. For inclusion criteria, we considered adults of both sexes, aged 18 to 59, with a diagnosis of obesity assessed by Body Mass Index (BMI) ≥ 30 kg/m^2^. Exclusion criteria included pregnancy; volunteers undergoing pharmacological treatment for body weight loss; use of corticosteroids or antiepileptic drugs; history of renal or cardiac disease; alcohol abuse; smoking; diagnosis of obesity secondary to genetic disorders; or the presence of eating disorders.

Data collection occurred between September 2021 and August 2023. After initial contact and the volunteer´s consent, an interview was conducted to provide information about the study, assess eligibility, and apply the anamnesis questionnaire. Subsequently, the volunteers were scheduled for the study protocol assessments, which included anthropometric measurements and nutritional evaluation.

Were included 77 individuals with severe obesity (BMI: 39.14 kg/m^2^ ± 5.57), of both sexes (77.90% female participants and 22.10% male), aged 36 ± 8 years, body mass of 106.2 kg (± 18.7), and height of 1.64 m (± 0.08).

This study was conducted according to the principles of the Declaration of Helsinki and was approved by the Research Ethics Committee of UNIFESP (CAAE: 36548020.0.0000.5505), with the Brazilian clinical trials registration number RBR-103k2gpz/ 2021. All procedures were explained to the volunteers, and they all provided their written consent.

#### Anthropometric measurements

All subjects’ body mass (kg) and height (m) were measured in the morning after a 12-hour fast and with them wearing minimum clothing, without shoes or accessories (such as watches or earrings). Body weight was assessed using the BOD POD^®^ Body Composition System (Life Measurement Instruments, Concord, CA), which has a maximum capacity of 250 kg and a sensitivity of 10 g. The equipment was calibrated before each assessment according to the manufacturer’s instructions. Height was measured using a Standard Sanny^®^ stadiometer (capacity up to 220 cm).

The body mass index (BMI) was calculated by dividing the weight by height squared (kg/m^2)^. For classification purposes, the cutoff points established by the World Health Organization ^(21)^ were used, defining class 1 obesity as BMI ≥ 30 kg/m^2^, class 2 obesity as BMI ≥ 35 kg/m^2^, and class 3 obesity as BMI ≥ 40 kg/m^2^. All assessments were conducted by the same researcher, who had been previously trained.

### Nutritional assessment

#### Dietary consumption assessment

Dietary consumption data were based on the mean of three 24-hour dietary recalls on non-consecutive days, with one day of the weekend. The 24-hour recalls were applied considering the multiple-pass method, consisting of: 1. Quick and uninterrupted listing of food and drinks; 2. Review of forgotten foods; 3. Time and occasion of food consumption; 4. Details and description of the quantities consumed; 5. Final review ^(22)^. Participants had access to reference photographs of utensils (such as spoons and cups) to assist in estimating portion sizes, with the aim of improving the accuracy of dietary data collection. The dietary data were analyzed using the Nutrabem^®^ software, which uses as its database the *Tabela Brasileira de Composição de Alimentos* (TACO) and the Database of Nutrients from the U.S. Department of Agriculture (USDA) ^(23)^. The classification of foods based on the extent and purpose of industrial processing was carried out using the NOVA classification system ^(4)^. All foods were listed and culinary preparations were evaluated individually. For homemade or artisanal preparations, the recipes were deconstructed into standardized ingredients by the software, and the components were listed. For preparations classified as industrial formulations, such as ready-to-eat meals, fast food, mass-produced packaged breads, and flavored yogurt, the labeling was analyzed, and the foods were entered without separating their ingredients; subsequently, all foods were allocated into groups according to their processing. Foods that did not fit perfectly into any category were classified in consensus with other researchers. Data collection for the 24-hour dietary recalls and the classification process according to the NOVA system was conducted by a trained researcher, following established guidelines ^(24)^.

The diet quality index was also assessed using the “Diet Quality Index associated with the Digital Food Guide (DQI-DFG)”, developed based on guidelines from the Department of Nutrition of the Harvard School of Public Health, with adjustments made to reflect the dietary habits of the Brazilian population. The index classifies foods into two groups: moderation components – foods that can be harmful if consumed in excess: sugar and sweets, dairy drinks and sweetened yogurts, processed meats and bovine and pork meat, refined grains and flours, some cereals and potatoes, crackers and snacks, and processed fat; and adequacy components – foods that are sources of nutrients and bioactive compounds: fruits and vegetables, leafy vegetables, cereals and whole grains, tubers and roots, poultry, fish and eggs, legumes, nuts, oils and fats, and dairy products. Each food group receives a score according to the portion consumed relative to a reference range. The final score ranging from 0 to 100: < 40 points is a low-quality diet; between 40 and 70 points, intermediate quality diet; and > 70 points, good quality diet ^(25)^.

#### Eating behavior assessment

Eating behavior was assessed using self-administered questionnaires, applied online through the Google Forms^®^ platform. The internal consistency of each questionnaire was evaluated using Cronbach’s α and McDonald’s ω coefficients. Values ≥ 0.70 were considered indicative of good internal consistency ^(26)^.

- **Bulimic Investigatory Test Edinburgh (BITE):** consisting of 33 items divided into two scales that assess symptoms and severity of bulimia or binge eating. The symptom scale comprises 30 questions (“yes”/“no”) designed to investigate the presence of bulimic symptoms. For items 1, 13, 21, 23, and 31, one point is assigned for a “no” response, whereas all other items are scored for “yes”. A total score ≥ 20 may suggest the presence of binge eating and bulimia nervosa, while intermediate scores between 10 and 19, suggest unusual eating behavior without all the diagnostic criteria for bulimia or binge eating, but should be investigated considering the risk of early stages of eating disorders. The severity scale includes three questions that assess the intensity and frequency of binge eating and purging behaviors. A score > 5 are considered significant, and scores ≥ 10 indicate high severity ^(27)^. The Brazilian version was translated by Cordás & Hochgraf (1993) ^(28)^. Cronbach’s α: 0.79 and McDonald´s ω: 0.82.
- **Dutch Eating Behaviour Questionnaire (DEBQ):** comprising 33 questions divided into three subscales that address restrained eating (10 questions), external eating (10 questions), and emotional eating (13 questions), subdivided into emotional eating in response to diffuse or clearly labeled emotions. The questions are answered on a 5-point Likert scale, ranging from never, rarely, sometimes, often, to very often. The mean score of each subscale is calculated. The subscale with the highest score represents the predominant eating style. The DEBQ was translated and validated for the Brazilian population by Moreira and cols. (2017) ^(29)^. Cronbach’s α: 0.91 and McDonald´s ω: 0.92.
- **The Three Factor Eating Questionnaire (TFEQ-21):** composed of 21 questions that assess cognitive restraint (6 questions), emotional eating (6 questions), and uncontrolled eating (9 questions). The response format varies from 1 to 4 points. Subsequently, the mean score of each evaluated eating behavior dimension was calculated and converted to a 0–100 scale. The higher score in an analyzed dimension indicates the predominance of that eating style. It was translated and applied to a Brazilian sample by Natacci & Ferreira Júnior (2011) ^(30)^. Cronbach’s α: 0.80 and McDonald’s ω: 0.82.

#### Statistical analyses and sample size

For statistical analysis, the STATISTICA program (StatSoft — South America; São Paulo — Brazil; version 7.0 for Windows) was used. The value considered for statistically significant change was set at α < 0.05. The normality of the data was assessed using the Shapiro-Wilk test. Parametric variables were expressed as mean ± standard deviation (mean ± SD), and non-parametric variables were expressed as median values (minimum and maximum). In group comparisons, one-way ANOVA tests were applied to BMI, symptom subscale (BITE), external and restrained eating subscale (DEBQ), cognitive restraint and uncontrolled eating subscale (TFEQ-21), followed by the application of the post hoc Tukey test when assessing parametric variables. Non-parametric variables, including body weight, severity subscale (BITE), emotional eating subscale (DEBQ) and emotional eating subscale (TFEQ-21), were analyzed using the Kruskal-Wallis and Mann-Whitney tests. Association analyses were performed using the Generalized Linear Model (GzLM), with the SPSS software version 21.0 (SPSS Inc., Chicago, Illinois, USA), and applying the appropriate distribution (gamma or linear) as determined by the Akaike Information Criterion (AIC). The p-values were reported along with β coefficients and confidence intervals.

For the sample size calculation, the G*Power 3.0.10 analysis program was used, adopting the multiple linear regression test. We considered a significance level of 5%, a test power of 95%, and an effect size of 0.15. The calculated sample size was 74 volunteers. Additionally, to determine the sample size, a previous study published with a similar sample to the one proposed in the present investigation was considered ^(27)^.

## RESULTS

Seventy-seven volunteers of both sexes were evaluated, with the majority being female (77.9%), with a mean age of 36 years (±8.7), body weight of 106.2 kg (±18.7), height of 1.64 m (±0.08), and a clinical diagnosis of class 2 obesity (BMI: 39.14 kg/m^2^ ±5.57). For the investigation of our outcomes, the sample was divided into three groups based on the tertile of ultra-processed food calorie intake (%), with the first tertile consuming less than 24.10% of total calories from ultra-processed foods (n = 25); the second tertile consuming between 24.10% and 35.40% (n = 26); and the third tertile consuming above 35.40% of calories from ultra-processed foods (n = 26). Considering the variables of body weight and BMI, no differences were observed between the evaluated groups.

The presence and severity of binge eating and bulimia symptoms were assessed using the Bulimic Investigatory Test Edinburgh (BITE) questionnaire. In the sample, 24.7% of the participants presented normal eating behavior scores, 51.9% had a medium score, suggesting the presence of unusual eating behavior, and 23.4% had high scores, indicating binge eating with possible presence of bulimia. Regarding the severity subscale, 46.8% showed no severity of symptoms, 40.3% had clinically significant symptoms, and 13% exhibited a high degree of severity.

Although the average of the tertile groups did not reach the cutoff point that suggests the presence of an eating disorder, all groups had scores consistent with unusual eating behavior. In the symptoms subscale, it was identified that the third tertile group had a higher score value when compared to the first tertile group (p=0.008). There was no difference between the groups based on the severity subscale (**Table 1**).

**Table 1.** Presence of binge eating and bulimia symptoms according to BITE, emotional, external, and restrained eating according to DEQB, and cognitive restraint, emotional eating, and uncontrolled eating according to TFEQ-21 in adults with obesity based on the tertile of ultra-processed food consumption

Variables	All (n = 77)	1^st^ tertile (n = 25)	2^nd^ tertile (n = 26)	3^rd^ tertile (n = 26)
Bulimic Investigatory Test Edinburgh				
Symptom subscale	14.50 ± 6.13	11.88 ± 5.75	14.62 ± 5.76	16.90 ± 6.01^A^
Severity subscale*	5 (0–22)	5 (0–14)	5 (0–18)	5.50 (0–22)
Dutch Eating Behaviour Questionnaire				
Emotional eating*	3.08 (1–5)	2.77 (1–4.69)	2.69 (1.23–4.62)	3.62 (2.46–5)^A,B^
External eating	3.28 ± 0.71	3.06 ± 0.69	3.10 ± 0.63	3.67 ± 0.66^A,B^
Restrained eating	2.78 ± 0.52	2.85 ± 0.52	2.90 ± 0.53	2.60 ± 0.46
The Three Factor Eating Questionnaire				
Emotional eating (0–100)*	61 (0–100)	56 (0–94)	44.50 (0–83)	75 (39–100)^A,B^
Cognitive restraint (0–100)	46.30 ± 14.30	48.60 ± 17.20	48.81 ± 11.39	41.90 ± 13.56
Uncontrolled eating (0–100)	46.60 ± 21.70	40.30 ± 21.80	42.38 ± 19.99	56.30 ± 20.26^A,B^

BITE: Bulimic Investigatory Test Edinburgh; DEBQ: Dutch Eating Behaviour Questionnaire; TFEQ-21: The Three Factor Eating Questionnaire.

A Statistical significance of the1^st^ tertile;

B Statistical significance of the 2^nd^ tertile;

* Non-parametric data expressed in median (minimum and maximum values); parametric data expressed in mean and standard deviation.

ANOVA with post hoc Tukey test (parametric data); Kruskal-Wallis test (non-parametric data).

When evaluating eating styles associated with restrained, external, and emotional eating using the Dutch Eating Behaviour Questionnaire (DEBQ), it was observed that 37.8% of the sample had higher scores for external eating, 36.5% for emotional eating, and 25.7% for restrained eating. The third tertile group showed higher values for emotional and external eating variables compared to the first tertile group (p = 0.005; p = 0.004, respectively) and the second tertile group (p = 0.0003; p = 0.007, respectively) (**Table 1**).

Furthermore, when analyzing factors associated with emotional eating, cognitive restraint, and uncontrolled eating according to the Three Factor Eating Questionnaire (TFEQ-21), it was identified that 52% had higher scores on the emotional eating subscale, 29.3% for cognitive restraint, and 18.7% for uncontrolled eating. It was observed that the third tertile group showed higher scores for emotional eating (first tertile p = 0.002; second tertile p=0.0009) and uncontrolled eating (first tertile p = 0.024; second tertile p = 0.048) when compared with the other groups (**Table 1**). For the other variables presented in **Table 1**, no significant differences were found between the groups investigated.

In the evaluation according to the Diet Quality Index–DQI-DFG, it was observed that the total sample presented a score intermediate quality diet (43 ± 10), as well as the first tertile (46.75 ± 8.72) and second tertile groups (45 ± 8). However, the third tertile group presented low-quality diet classification (37 ± 10), with a significant difference when compared to the first and second tertile groups (p = 0.001; p = 0.003, respectively) (**Table 2**).

**Table 2.** Nutritional profile according to the tertile of ultra-processed food consumption in adults with obesity

Variables	All (n = 77)	1^st^ tertile (n = 25)	2^nd^ tertile (n = 26)	3^rd^ tertile (n = 26)
DQI-DFG	43.08 ± 10.17	47 ± 9	46 ± 9	37 ± 10^A,B^
Total caloric intake (kcal)*	1661 (756–4774)	1667 (756–2623)	1432 (1001–2312)	2114 (1139–4774)^B^
Unprocessed foods (% kcal)	49 ± 14	60 ± 14	52 ± 8^A^	37 ± 11^A,B^
Processed culinary ingredients (% kcal)*	7 (1–33)	8 (3–33)	8 (3–17)	4 (1–13)^A,B^
Processed foods (% kcal)	11 ± 8	14 ± 9	9 ± 5	9 ± 7
ultra-processed foods (% kcal)	32 ± 15	17 ± 7	30 ± 3^A^	49 ± 11^A,B^
Protein (%)	20 ± 4	22 ± 4	21 ± 4	17 ± 3^A,B^
Fat (%)	32 ± 5	32 ± 5	32 ± 5	23 ± 11
Carbohydrate (%)	48 ± 7	47 ± 7	49 ± 8	49 ± 6

DQI-DFG: Diet Quality Index associated with the Digital Food Guide. Cut-off points: < 40 points: low quality diet; 40-70 points: intermediate quality diet; > 70 points: good quality diet;

A Statistical significance of the1^st^ tertile;

B Statistical significance of the 2^nd^ tertile;

* Non-parametric data expressed in median (minimum and maximum values); parametric data expressed in mean and standard deviation.

ANOVA with post hoc Tukey test (parametric data); Kruskal-Wallis test (non-parametric data).

In the analysis of dietary intake, the median caloric consumption was 1661 kcal (756.07–4774.40), and the distribution according to the NOVA classification of 48% (±14) of unprocessed or minimally processed foods, 7% (1–33) of processed culinary ingredients, 11% (±8) of processed foods, and 32% (±15) of ultra-processed foods (**Table 2**).

Analyzing dietary intake, there was a difference in total caloric consumption between the second and the third tertile groups (p = 0.0002). In the evaluation of the NOVA classification, the first tertile group showed higher consumption of unprocessed and minimally processed foods compared to the second (p = 0.002) and the third tertiles (p = 0.0001). There was also a difference between the second and the third tertiles (p = 0.0001). Additionally, the first and the second tertile groups had a higher proportion of processed culinary ingredients compared to the third tertile group (p = 0.0005 and p = 0.0006, respectively) (**Table 2**).

In the consumption of ultra-processed foods, it is possible to observe that the third tertile group had a higher contribution of these foods to caloric intake compared to the first (p = 0.0001) and the second tertile groups (p = 0.0001). The second tertile group also had a higher intake when compared to the first tertile group (p = 0.0001) (**Table 2**).

Considering the assessment of macronutrient intake, the average protein intake in the total sample was 20% (±4), lipid intake was 32% (±5), and carbohydrate intake was 48% (±7). In the analysis separated by tertile, it was observed that the third tertile group had a lower total protein intake compared to the first (p = 0.0001) and the second tertile groups (p = 0.001). There was no difference in the average intake of total lipids and carbohydrates between the groups (**Table 2**).

The association analysis reinforces the previous findings. It was observed that the consumption of ultra-processed foods was positively associated with the symptom subscale of binge eating and bulimia (p = 0.018, β = 0.008, 1.001 – 1.015 95% IC). Additionally, a positive association was found between ultra-processed food consumption, emotional eating (p = 0.001, β = 0.008, 1.003 – 1.014 95% IC) and external eating (p = 0.001, β = 0.005, 1.002 – 1.008 95% IC) according to the DEBQ; as well as with emotional eating (p = 0.008, β = 0.007, 1.002 – 1.013 95% IC) and uncontrolled eating (p = 0.006, β = 0.006, 1.002 – 1.010 95% IC), as assessed by the TFEQ-21. We also identified that the higher consumption of ultra-processed foods was associated with lower Diet Quality Index scores (p < 0.001, β = -0.008, 0.988 – 0.995 95% IC) (**Table 3**).

**Table 3.** Association between the presence and severity of binge eating and bulimia symptoms according to BITE, eating styles according to DEQB and TFEQ-21, Diet Quality Index (DQI-DFG) and consumption of ultra-processed foods (%)

Dependent variable	β	Wald	Exp (B)	(95% CI)	p-value
Bulimic Investigatory Test Edinburgh					
Symptom subscale	0.008	5.567	1.008	(1.001–1.015)	**0.018**
Severity subscale	0.009	2.858	1.009	(0.999–1.020)	0.91
Dutch Eating Behaviour Questionnaire					
Emotional eating	0.008	10.355	1.008	(1.003–1.014)	**0.001**
External eating	0.005	11.997	1.005	(1.002–1.008)	**0.001**
Restrained eating	-0.003	6.789	0.997	(0.994–0.999)	0.09
Three-Factor Eating Questionnaire					
Emotional Eating	0.007	7.005	1.007	(1.002–1.013)	**0.008**
Cognitive Restraint	-0.002	3.402	0.998	(0.995–1)	0.065
Uncontrolled Eating	0.006	7.627	1.006	(1.002–1.010)	**0.006**
Diet Quality Index	-0.008	22.435	0.992	(0.988–0.995)	**0.000002**

BITE: Bulimic Investigatory Test Edinburgh; DEBQ: Dutch Eating Behaviour Questionnaire; TFEQ-21: The Three Factor Eating Questionnaire; DQI-DFG: Diet Quality Index associated with the Digital Food Guide.

Generalized Linear Model (GzLM). The consumption of ultra-processed foods (%) was used as the independent variable. Statistical significance p ≤ 0.05.

## DISCUSSION

The results of this study suggest positive association between higher consumption of ultra-processed foods and the presence of binge eating and bulimia symptoms, as well as higher scores for emotional eating, external eating, and uncontrolled eating. We also find that the group of volunteers with a higher consumption of ultra-processed foods exhibited higher scores for unusual eating behavior.

The high availability and easy access to ultra-processed foods may contribute to the worsening of obesity, not only due to its caloric content but also its potential neurobiological and metabolic effects, exacerbating symptoms of eating disorders and unusual eating behavior ^(19)^. So far, few studies have investigated the possible association between the consumption of ultra-processed foods and eating disorders in adults ^(18,31)^. In previous investigations, the analysis of data from a cohort of French adults identified that an increase in the consumption of ultra-processed foods was associated with a 21% higher risk of Binge Eating Disorder (BED) and 8% of bulimia nervosa (BN) ^(18)^.

To our knowledge, there have been no previous studies examining the consumption of ultra-processed foods and symptoms of binge eating and bulimia in a clinical sample of adults with obesity. Our findings support the hypothesis that the consumption of ultra-processed foods may negatively impact the eating behavior of individuals with obesity.

We observed a higher prevalence of women in our sample, which is consistent with studies showing that women are more likely than men to seek clinical and surgical treatment for obesity ^(32,33)^.

In the investigated sample, 23.4% of volunteers presented scores consistent with binge eating symptoms with probable presence of bulimia, and unusual eating behavior was observed in 51.9% of the individuals assessed. In a systematic review study that evaluated the presence of eating disorders in the general population, the prevalence of BED was found to be 2.3% for women and 0.3% for men, while the prevalence of bulimia was 1.5% for women and 0.1% for men ^(34)^. In individuals with obesity, the prevalence of BED can vary from 9.5% to 40.6% ^(35)^, corroborating our results.

Our findings are similar to the study by Erden Aki and cols. (2022) ^(36)^, who evaluated 465 patients with obesity who were candidates for bariatric surgery. In their study, 23.6% of the investigated sample achieved scores indicative of binge eating, and 57.8% presented unusual eating behavior according to the BITE instrument. In the analysis of tertile groups, volunteers with a higher consumption of ultra-processed foods had higher scores for unusual eating behavior. The consumption of palatable foods can impair eating control and produce behaviors similar to dependence on these foods, due to the high fat and sugar content, which leads to excessive consumption and worsening obesity ^(37)^.

Non-homeostatic control of food intake, also referred to as “hedonic hunger”, is defined as food consumption in the absence of physiological hunger signals, driven by pleasure, and is accentuated in individuals with obesity compared to individuals without obesity. This behavior is mediated by environmental and cognitive factors, such as learning and memory, and is influenced by the action of brain’s reward systems through dopamine release, being activated by the intake of palatable foods with high sugar and fat content, such as ultra-processed foods ^(38-41)^.

Eating behavior patterns of individuals are known as eating styles and can be divided into three dimensions: restrained eating, characterized by intentional food restriction aimed at body weight control; external eating, when the presence of food can trigger excessive food intake; and emotional eating, in which emotional components such as stress, fear, anxiety, and even depression can influence food choices and preference for palatable foods, especially those with high sugar content ^(42-44)^.

In our study, it was observed that individuals with a higher consumption of ultra-processed foods had higher scores for emotional and external eating. In the study conducted by Erden Aki and cols. (2022) ^(36)^, a similar result was observed, in which participants had higher average scores for the emotional and external eating subscales. Corroborating, a study with 121 participants with obesity, Benzerouk and cols. (2020) ^(45)^ found that participants with binge eating symptoms exhibited elevated emotional and external eating, suggesting that individuals with binge eating tendencies may use palatable foods to compensate for negative feelings or to cope with distress. In addition, in our study, it was found that volunteers in the third tertile of ultra-processed food consumption scored higher in the emotional eating domains than the others; additionally, in the food disinhibition subscale, individuals in the third tertile scored higher than those in the first and second tertiles.

Aymes and cols. (2022) ^(46)^ evaluated 2,237 patients with obesity and 403 participants without obesity. Through the application of the TFEQ-21, it was identified that all three scores of eating behavior were higher in individuals with obesity compared to the control group. Additionally, the authors observed that dimensions of food disinhibition and emotional eating were associated with obesogenic eating habits, such as eating at night or in front of the television or stopping eating due to gastric fullness compared to a feeling of satiation. Moreover, in the study by Jeffers and cols. (2019) ^(47)^, the presence of a negative effect, such as emotional eating, increased susceptibility to hunger, and eating disinhibition, was associated with a higher likelihood of consuming foods high in sugar. Improvement in emotional eating behavior may contribute to the body weight loss process ^(48)^.

Considering the assessment of dietary intake, the average caloric contribution of unprocessed and minimally processed foods in the total sample was 49% ± 14, and for ultra-processed foods, it was 32% ± 15. Although the consumption of unprocessed foods is similar to that of the Brazilian population (53.25%), the intake of calories from ultra-processed foods exceeds the population’s consumption (19.69%) ^(49)^. In an analysis of a community sample from Rio de Janeiro, Brazil, the consumption of hyperpalatable foods by volunteers with BED was 63%, and for BN, it was 44% ^(50)^. In individuals with obesity, Lopes Pinto and cols. (2019) ^(51)^ identified that the consumption of ultra-processed foods was 27.2%, while unprocessed and minimally processed foods accounted for 55.7% of daily calory intake.

The high consumption of ultra-processed foods is related with a low-quality diet, characterized by high intake of total and saturated fats, low intake of proteins, fibers, and micronutrients such as magnesium, potassium, zinc, and vitamins C, D, B12, and niacin ^(52)^. In an analysis of data from the *Inquérito Nacional de Alimentação* (INA — National Food Survey) 2017–2018 ^(53)^, the recommended fruits and vegetables consumption (≥ 400g/day) was observed in only 12.9% of respondents. In accordance with previous studies, our research found an association between the consumption of ultra-processed foods and low diet quality.

In the assessment of dietary macronutrient intake considering the NOVA classification system, volunteers with a higher consumption of ultra-processed foods showed a lower intake of unprocessed and minimally processed foods in their diet. They also had a lower total protein intake. Corroborating our findings, in the study by Martini and cols. (2021) ^(52)^, the consumption of ultra-processed foods was inversely correlated with the total protein intake in the highest tertile. For each one point increase in the energy contribution from ultra-processed foods, a reduction of 0.07% in total protein intake was observed. A similar result was identified in the study by Bielemann and cols. (2015) ^(54)^, in which there was a tendency to reduce daily protein intake with increasing consumption of ultra-processed foods. In a clinical trial in which volunteers were subjected to both an ultra-processed and an unprocessed diet, an increase in carbohydrate and lipid intake from ultra-processed foods was observed, but not in protein intake. There was a slight decrease in protein intake compared to the unprocessed diet ^(55)^.

Reinforcing our findings, data from the *Pesquisa de Orçamentos Familiares* (POF – Consumer Expenditure Survey) 2008–2009 ^(56)^ indicate an inverse correlation between the acquisition and consumption of vegetables and of ultra-processed foods, demonstrating the ability of ultra-processed foods to replace the intake of healthier foods. The consumption of fruits and vegetables can reduce the risk of diseases, such as cardiovascular disease, coronary heart disease, stroke, cancer, and all-cause mortality due to the presence of fiber, micronutrients, e.g., vitamins and minerals, and bioactive compounds, such as polyphenols ^(57)^. The consumption of these foods also impacts mental health, reducing the risk of psychological distress and depression ^(58)^. Reinforcing the benefits of consuming whole foods for health, the World Health Organization (WHO) ^(59)^ recommends a daily intake of ≥ 400g or five servings of fruits and vegetables per day.

We recognize that this study has certain limitations. Although our findings suggest a possible association between ultra-processed food consumption and eating disorder symptoms, reinforcing previous research, the cross-sectional design and the use of a clinical sample from a single urban center limit the extrapolation of the results to the general population. Future longitudinal studies involving more diverse samples could strengthen these results.

In the dietary intake analysis, the use of the mean of 24-hour dietary recalls, although widely used, may be considered a limitation, as it does not completely eliminate intra-individual variability and may underestimate or overestimate actual food intake, especially among individuals with obesity. Furthermore, the use of self-reported data, both in eating behavior questionnaires and the dietary recalls, may not accurately reflect reality due to potential biases such as social desirability and volunteers’ memory recall.

However, the questionnaires demonstrated good internal reliability and the application of the instruments was conducted with the aim of minimizing this effect. Additionally, the instrument was not validated for the NOVA classification system, requiring researcher’s to accurately perform the classification.

In conclusion, based on our findings, more than half of the individuals with obesity presented unusual eating behavior, with the group of volunteers showing higher consumption of ultra-processed foods also displaying higher scores for unusual eating behavior, emotional eating, external eating, and uncontrolled eating. Regarding dietary intake, these volunteers had low-quality diet and lower total protein intake.

Therefore, we suggest that the treatment of individuals with obesity should consider diet quality along with the eating behavior dimensions that may reinforce unhealthy food choices. In this context, it is essential to encourage the consumption of unprocessed and minimally processed foods and to reduce the intake of ultra-processed foods, in addition to establishing public policies that promote healthier food systems and access to adequate nutrition. These policies should include incentives for the production and distribution of healthy foods, the regulation of ultra-processed food advertising, and clear nutritional labeling, which may support healthier food choices.

## Data Availability

datasets related to this article will be available upon request to the corresponding author.
